# Automated documentation of vital parameters in wards using portable stations - Effect on proper triggering of the rapid response team: a study protocol of a cluster randomized clinical trial

**DOI:** 10.5935/0103-507X.20220101-en

**Published:** 2022

**Authors:** José Cesar Ribeiro, Cristina Sgorbissa, Karla Aparecida Silva, Maria de Lourdes Dias Braz, Ana Clara Peneluppi Horak, Marina Lazzari Nicola, Rodrigo Magalhães Gurgel, Samira Martins Tokunaga, Karina Leal Negrelli, Gabriela Souza Murizine, Fernando Medrado Júnior, Rita de Cassia Pires Coli, Alexandre Biasi Cavalcanti, Aline Marcadenti

**Affiliations:** 1 Department of Nursing, HCor-Hospital do Coração - São Paulo (SP), Brazil.; 2 Research Institute, HCor-Hospital do Coração - São Paulo (SP), Brazil.

**Keywords:** Vital signs, Medical records systems, computerized, Nursing stations, Hospital rapid response team, Health information interoperability

## Abstract

**Objective:**

To evaluate the effectiveness of the Welch Allyn Connex^®^ Spot Monitor/Hillrom Connecta™ solution in activating the rapid response team in a timely manner compared to manual activation.

**Methods:**

The Hillrom study is a single-center, open-label, superiority, cluster-randomized, parallel-group (1:1 allocation ratio) clinical trial that will be conducted in a tertiary hospital. Two sets of three wards with 28 beds will be included (one as the intervention cluster and the other as the control). The wards will be randomly assigned to use the Welch Allyn Connex^®^ Spot Monitor/Hillrom Connecta™ automated solution (intervention cluster) or to maintain the usual routine (control cluster) regarding rapid response team activation. The primary outcome will be the absolute number of episodes of rapid response team triggering in an appropriate time; as secondary outcomes, clinical features (mortality, cardiac arrest, need for intensive care unit admission and duration of hospitalization) will be assessed according to clusters in an exploratory way. A sample size of 216 rapid response team activations was estimated to identify a possible difference between the groups. The protocol has been approved by the institutional Research Ethics Committee.

**Expected results:**

The Welch Allyn Connex^®^ Spot Monitor/Hillrom Connecta™ automated solution is expected to be more effective in triggering the nurse call system to activate the rapid response team in a timely and adequate manner compared to manual triggering (usual practice).

**ClinicalTrials.gov:**

NCT04648579

## INTRODUCTION

Clinical decisions are based on data, which must be accurate and collected in a timely manner.^(1)^ In hospital settings, timely information is essential to trigger faster responses that can ultimately determine the patient’s clinical outcome.^(2,3)^

Hospitalized individuals have their vital signs collected periodically with the aim of preventing clinical deterioration, which could potentially reduce several patient-centered outcomes.^(4)^ Vital sign measurement practices vary greatly due to different risk profiles, clinical complexity, and local protocols, among other factors. Data collection is often manual, requiring computer entry of readout values or traditional pen and paper workflows, which can result in errors and deviations in care.^(5)^ Manual recording can also delay triggering specific protocols for the deteriorating patient, especially rapid response teams (RRTs).

Despite having a crucial role in hospital care,^(6)^ data regarding the benefits of RRTs on clinical outcomes are still controversial. Metanalyses from longitudinal studies (cohort and interventional studies) have shown that RRT activation may lead to little or no difference in hospital mortality, unplanned intensive care unit (ICU) admissions, length of hospital stay or adverse events; in addition, the quality of evidence for these outcomes was low or very low.^(7,8)^ Large and well-designed clinical trials for evaluating the impact of RRTs properly triggered on relevant features are still necessary.

Automated devices that can collect and exchange information without human interference have been emerging as an interesting alternative to manual data collection.^(9)^ These devices can also trigger specific protocols, such as RRTs, without direct human interference.^(4,9,10)^ However, despite the potential benefits of automated devices over manual data collection,^(11,12)^ few clinical studies have been performed to directly compare the two methods with respect to triggering the RRT and hospital outcomes.^(7,9)^ Therefore, we designed a randomized clinical trial to assess the hypothesis that the use of an automated vital signs monitoring system associated with automatic activation of the RRT can result in an increase in the number of faster and more effective activations in an appropriate timely manner.

### Objectives

The primary objective of the study is to evaluate the effectiveness of the solution Welch Allyn Connex^®^ Spot Monitor (CSM)/Hillrom Connecta™ on triggering the RRT in an appropriate timely manner compared to manual triggering.

The secondary objectives are to assess the following in an exploratory way:

- Clinical outcomes (mortality, cardiac arrest, need for ICU hospitalization, and duration of hospitalization) among patients who had the RRT automatically triggered by the Welch Allyn Connex^®^ Spot Monitor (CSM)/Hillrom Connecta™ compared to patients who had the RRT triggered manually
- The effects of the Welch Allyn Connex^®^ Spot Monitor (CSM)/Hillrom Connecta™ on clinical outcomes (mortality, cardiac arrest, need for ICU hospitalization, and duration of hospitalization) compared between the intervention and the control wards

### Trial design

The Hillrom study (ClinicalTrials.gov Identifier: NCT04648579; 4th protocol version [13/Apr/2021]) is a national, unicentric, cluster, parallel-group, open-label, and superiority randomized clinical trial (allocation ratio 1:1).

## METHODS

### Study setting

**Figure 1:**
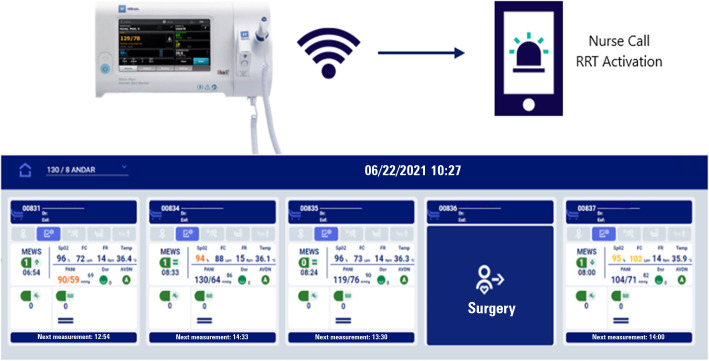
Connex^®^ Spot Monitor.

The study will be conducted in a tertiary hospital located in the Southeast region of Brazil. The HCor Research Institute (IP-HCor) will be responsible for the protocol and coordination of the study. Data collection will commence after compliance with all regulatory requirements, as well as training and adjustments to the platforms and communication network necessary for automatic activation of the RRT. There is no predetermined time for conducting the study; the admissions of patients to the inpatient units will determine the study length. Wards with a similar patient profile and a higher number of RRT activations compared to regular wards will be selected and randomly assigned to use the Welch Allyn Connex^®^ Spot Monitor (CSM)/Hillrom Connecta™ solution (Figure 1) or to maintain their usual routine.

This is portable equipment used to capture vital signs and calculate the early warning score to inform the care team about the risk of patient clinical deterioration at the bedside. The data are sent via a wireless network to the Hillrom Connecta™ platform to be visualized through a panel in the nursing station, and at the same time, the Connex® Spot Monitor triggers the rapid response team through the nurse call for priority care according to institutional triggering criteria.

RRT - rapid response team.

### Eligibility criteria

The inclusion criteria used to define the inpatient units (clusters) of the study will be availability as defined by the heads of the hospital’s nursing area. Patients admitted to the selected wards (all must be of medium complexity) who triggered the RRT during the recruitment period will be included in the study; patients with indications for blood pressure measurement via the lower limbs (ankle) and those who are not candidates for either resuscitation or organ support in the ICU will be excluded.

### Intervention

#### Description of the intervention

At the hospital where the study is conducted, current practice is based on triggering the RRT if any of the following signs are identified in a given patient-health care provider encounter:

- Decreased acute oxygen saturation to < 90%.
- Hypoglycemia (defined as capillary blood glucose < 50mg/dL).
- Change in respiratory rate (RR) to < 8rpm or > 28rpm.
- Systolic blood pressure (SBP) < 90 or > 200mmHg.
- Oliguria (defined by diuresis < 300mL in 24 hours).
- Heart rate (HR) < 40bpm or > 130bpm.
- Sepsis research, defined according to international guidelines.^(13)^
- Employee concern about the general status of the patient.
- Acute neurological deficit.
- Chest discomfort/pain.
- Modified Early Warning Scores (MEWS) ≥ 5.^(14)^

The proposed intervention involves an automated vital signs documentation system. This system consists of a portable medical device for measuring vital signs (Welch Allyn Connex^®^ Spot Monitor (CSM), Baxter International Inc, Deerfield, USA), which collects and analyzes the data acquired at the bedside to be sent later to a remote data processing point (Digital Control Station - DCS) using Hillrom Connecta™ software (Baxter International Inc, Deerfield, USA). Data on blood pressure, HR, temperature, and oximetry are measured at the bedside by the equipment. Other information, such as pain scores, RR and level of consciousness, are manually collected by the nursing team and entered into the Welch Allyn Connex^®^ Spot Monitor (CSM). After collecting all the data, an institutionalized protocol for the Early Warning Score (EWS) protocol already configured into the monitor calculates the final score for patient deterioration risk based on the hospital criteria. All results are automatically stored and transferred to the DCS, and they are sent to the Hillrom Connecta™ software platform. The vital signs and the final EWS can be visualized through a panel at the nurses’ station.

In addition, if at least one of the criteria for activating the RRT is identified, the Hillrom Connect™ solution triggers the nurse call system that automatically activates the RRT.

In the control wards, the usual practice will be maintained, which comprises entering data into an Excel^®^ spreadsheet and maintaining the frequency of monitoring/measurements according to the MEWS. The same criteria for activating the RRT will be used; if any are identified by the health professional, they will trigger the RRT manually at the bedside. The RRT has up to 5 minutes to attend the patient.

#### Criteria for discontinuing or modifying the intervention

Considering that the interventions proposed in this study will be applied on the wards and not directly on the patients, who will receive all care in a standardized way regardless of the inpatient unit to which they are admitted, there is no provision for discontinuity or modification of the intervention.

#### Strategies to improve adherence to the intervention

All professionals involved in the research will be trained in this protocol and in the use of the Welch Allyn Connex^®^ Spot Monitor (CSM) and Hillrom Connecta™. The IP-HCor investigators will contact the care team on a weekly basis to resolve doubts about the protocol and handling of the equipment. Retraining will be carried out as requested by the care team or upon identification of frequent failures by the investigators.

#### Outcomes

The primary outcome of the study will be the absolute number of episodes of RRT triggering in a timely manner, defined by any triggering that occurred in the units randomized to the study and for which the patient had critheria within a 24-hour window. Appropriate time will be considered if the RRT appears at the bedside within 5 minutes after the yellow code is triggered by a health professional. The triggering time as well as the arrival time are recorded by the care team on a specific form attached to the patient’s medical record.

The identification of whether the yellow codes were activated properly will be verified by a care team not participating in the study. This verification will be carried out by checking the codes identified by a specific platform provided by the company *Eritel Telecomunicações Ltda.* (Eritel Telecommunications Limited) *versus* the codes recorded in the medical record. If the platform originates a code, but it was not confirmed/effective according to the nursing team’s verification, it will be considered false triggering (improper triggering).

The following secondary outcomes will be considered: mortality rates, cardiac arrest, need for ICU hospitalization (defined according to institutional protocols and trained staff) and duration of hospitalization during the study.

#### Timeline

**Figure 2:**
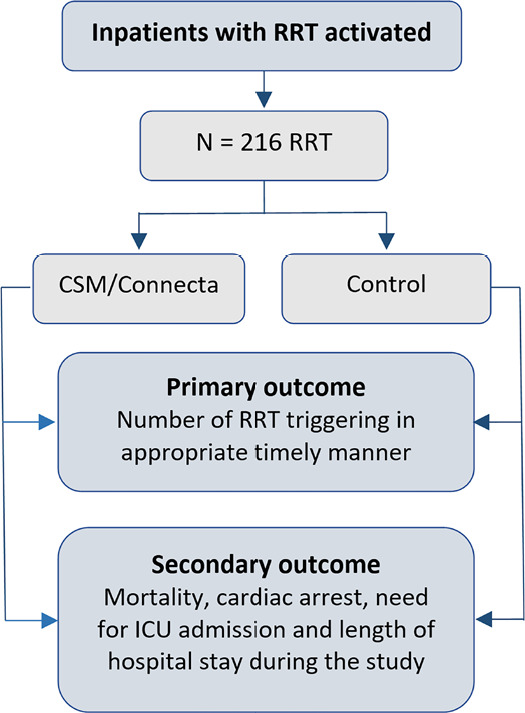
Study flowchart.

All RRT activations that occur in randomized units (intervention and control) will be recorded until the necessary number estimated in the sample calculation is obtained. The medical records of the respective patients will be obtained for evaluation and recording of data referring to primary and secondary outcomes (Figure 2). The general protocol schedule is described in table 1.

**Table 1:** General study schedule

	Study period			
	**Prerecruitment**	**Recruitment**	**Post-recruitment**	**Close-out**
Timepoint	November 2019 - March 2021	April - September 2021	October 2021 - March 2022	April - August 2022
Study design	X			
Submission and approval of the study protocol by the REC	X			
Team training	X			
Recruitment - Intervention group and control group		X		
Input of all data in the database (REDCap system)			X	
Data analysis				X
Submission of results				X

RRT - rapid response team; CSM/Connecta - Welch Allyn Connex^®^ Spot Monitor/Hillrom Connecta™; ICU - intensive care unit.

REC - Research Ethics Committee.

#### Sample size calculation

A set of three wards with 28 beds will be considered the intervention cluster (with the Welch Allyn Connex^®^ Spot Monitor (CSM)/Hillrom Connecta™ solution), and a set of three wards with 28 beds will be considered the control cluster. The average monthly number of episodes of RRT triggering in the six selected wards is 12, of which it is estimated that approximately 70% are triggered in a timely manner. For an absolute increase of 18% in the proportion of timely RRT triggering (88%) in the intervention group, with a test power of 90% and a significance level of 5%, we estimated a sample size of 216 RRT activations.

#### Recruitment

The care team will be responsible for surveying all RRT triggering that occurs in the randomized units. This survey is carried out using the institution’s own printed form, as well as the collection of data recorded on a platform provided by the company *Eritel Telecomunicações Ltda*.

The information regarding triggering will be provided to IP-HCor for registration and subsequent data collection from the medical records. In the medical record, if any code generated by the platform is not identified, it will be identified as a false triggering.

#### Allocation

The allocation of the selected wards to the intervention or control groups will be performed randomly and stratified by blocks according to the size of the cluster through the *sample* function of R 4.0.2 software (R Core Team, Vienna, Austria, 2020). Only the IP-HCor statistician team will have access to the allocation list.

#### Blinding

Considering the nature of the intervention, this will be an open-label study in which the researchers, the care team and the patients will be aware of the control or intervention clusters. During statistical analyses, investigators and statisticians will be blinded to the study groups. To avoid contamination between the groups as much as possible, the work schedules of individual workers and the shifts of the nursing teams will undergo the least possible change.

#### Data collection methods

In addition to the data regarding the recruitment period, information will be collected to describe the characteristics of the clusters and patients hospitalized in the selected wards for a period of three months prior to the beginning of the protocol: January 2021, February 2021 and March 2021. The institution’s Department of Epidemiology will provide the following variables from institutional records (if available).

#### Cluster-level data

1. Baseline variables (at the time of hospitalization): age, sex, type of hospitalization (surgical or clinical).
2. Outcomes (during hospitalization): number of RRTs triggered, yellow code rates, in-hospital mortality, duration of hospitalization, need for ICU hospitalization, and number of cardiac arrests.

We will also present the total number of RRTs triggered in this period (at the hospital level) and the number of hospital admissions on the selected wards both in this period and during the study.

#### Individual-level data

1. Baseline variables (at the time of hospitalization): age, gender, type of hospitalization, Charlson morbidity score.^(15)^
2. Outcomes (during hospitalization): in-hospital mortality, duration of hospitalization, need for ICU hospitalization, cardiac arrest, and selected relevant clinical events (stroke, myocardial infarction, and sepsis).

The nursing teams that work in the selected units will receive training prior to the beginning of the clinical trial. During the training, the study protocol and the Good Clinical Practices (GCP) guide will be presented. Hands-on training in the handling of the device by a device specialist will be provided to the care team at selected facilities that will use both Welch Allyn Connex^®^ Spot Monitor (CSM) and Hillrom Connecta™ devices.

Data will be collected through the physical records of patients who require the triggering of the RRT code in the selected wards during the recruitment period. These data will comprise age, sex, date of admission, reason for hospital admission, diagnostic class of hospital admission, previous comorbidities, date and time of yellow code triggering, physiological data in the last 24 hours (HR, RR, SBP, diastolic blood pressure, temperature, oxygen saturation, level of consciousness, capillary blood glucose [if available], chest discomfort/pain, leukocytes [if available], presence of sepsis, diuresis, poor general condition of the patient) collected at four time points, problems related to yellow code triggering, clinical outcomes, and hospital discharge/transfer/death. The frequency of vital signals obtained will be the same between groups (four measurements 24/7 in each group).

The analysis of the physical record and the input of data for the electronic case report form (CRF) will be carried out by HCor professionals, who will be previously trained in relation to the data capture system.

#### Data management

Data collection will be carried out through electronic CRF in the Academic REDCap environment. Data are entered directly into the data capture system by team members from the coordinating center, as there is still no communication between the Welch Allyn Connex^®^ Spot Monitor (CSM)/Hillrom Connecta™ solution and the REDCap system. All episodes of RRT triggering will be confirmed by the care team, as false RRT triggering may occur unduly or accidentally during the study period. The sponsor will support and maintain the devices and software throughout the period of use in the clinical trial. All nurse staff from the intervention group will be trained to trigger a RRT manually in case the system fails; in this case, this activation will be registered and dealt with as an intention to treat.

Data monitoring will be carried out by a data management team to collect *missing* data and inconsistencies using R software. Once all data are entered into the system and all discrepant or missing data are resolved, the statistician team will review and lock the database for further statistical analysis.

#### Statistical analysis

The demographic and clinical characteristics of the sample will be summarized according to the groups in absolute and relative frequencies for the categorical variables; continuous variables will be presented by position statistics (mean, median) and scale (standard deviation and interquartile ranges).

The analysis for the primary outcome (events that trigger the RRT in a timely manner) will be conducted with a logistic regression model considering binomial distribution from generalized estimation equations, considering a uniform work correlation matrix between patients of the same ward adjusted for the baseline number of episodes of RRT triggering in an appropriate timely manner. Other outcomes will be compared using a similar methodology considering the response distribution that best fits the data. All results will be presented considering measures of effect with respective 95% confidence intervals.

Sensitivity analyses for the primary outcome considering time series assessments and time of RRT activation (weekday *versus* weekend; night *versus* day shifts) will be performed comparing treatment groups. The results will be presented in graphs with monthly indicators.

It is not expected that there will be a large amount of missing data. However, if some of the primary and secondary outcomes are missing, the missing data rate will be reported by group, and the values will be imputed by chained equation multiple imputation methods using the mice package with sample base characteristics.

All analyses will be performed with statistical R software 4.0.2 (R Core Team, Vienna, Austria, 2020). Interim analyses or the participation of a Data and Safety Monitoring Committee (DSMC) are not foreseen in the protocol.

#### Ethical issues

This study was approved by the Research Ethics Committee (REC) of HCor under CAAE no. 26298019.4.0000.0060. Amendments and specific changes to the protocol will be carried out according to its progress and duly forwarded to the institutional REC (previous versions: 1st version - November 2019; 2nd version - September 2020; 3rd version - November 2020). Audits are not planned for this protocol; however, the sponsor may require information and reports during the conduct of the clinical trial and after its completion.

Considering that the collection and evaluation of the study variables as well as the triggering of RRTs are routines in clinical care practice and that the data will be collected through medical records, the REC-HCor was asked to waive the Free and Informed Consent Form for this research protocol. However, the investigators obtained institutional authorization to carry out the same.

There is minimal risk of loss of confidentiality associated with the study. The risk will be minimized by using traditional precautions for the storage of paper records and electronic records. Patient identifiers will not be used in reports or publications of this study.

#### Dissemination policy

After the publication of the results, we will disseminate the study to the entire care team of the participating center and to the sponsor through face-to-face and/or virtual presentations. We will also present the results at important congresses and events in the area.

#### DISCUSSION AND TRIAL STATUS

The measurement of vital signs is a fundamental component of patient assessment, providing the basis for clinical decision-making from treatment to hospital discharge. Therefore, these data must be accurate and quickly accessible so that safe decisions can be made.^(16)^ In an American university hospital, error rates were evaluated for electronic documentation of vital signs compared to manual records on paper. As a result, it was found that the use of the system reduced vital signs recording errors by more than half compared to traditional manual documentation (error rates: 4.4% and 10%, respectively).^(17)^ In addition, the implementation of the automated clinical documentation system allowed the nursing team to increase the time spent on direct patient care.^(18)^

Despite the lack of consistent evidence showing the effectiveness of RRT systems on clinical outcomes, they have been implemented at hospitals worldwide.^(6)^ After the introduction of RRTs in a large Brazilian nonprofit hospital, a significant reduction in waiting time for ICU beds among inpatients who could not be admitted immediately after indication was determined, as well as an increase in the recognition of palliative care patients; however, no difference in hospital mortality was detected.^(19)^ Similar results were observed after the implementation of RRTs in a Brazilian university hospital, where a reduction was observed in in-hospital cardiac arrest but not in hospital mortality.^(20)^

Electronic systems for automated notification of vital signs may contribute to reducing call delays, one of the most important barriers to successful implementation of RRTs^(21)^ associated with increased hospital mortality.^(22)^ In addition, they may increase the number of RRT activations. In a before-and-after study conducted in wards in the UK, the use of an electronic vital signs monitoring solution increased RRT activations from 405 to 524 (p = 0.001). In addition, a decrease in overall mortality and in the number of cardiac arrests was observed during the protocol intervention period.^(23)^ However, it is noteworthy that before-and-after studies are susceptible to a number of methodological biases compared to randomized trials, which can ultimately invalidate the results or impair the clinical significance of the study.^(24)^

The Hillrom study aims to evaluate, through a cluster randomized clinical trial, the effectiveness of an automated vital signs monitoring system associated with the automatic activation of the RRT on the absolute number of triggers in an appropriate timely manner. Additionally, we will assess clinical outcomes (mortality, cardiac arrest, need for ICU hospitalization and duration of hospitalization) in an exploratory manner according to the study groups. The assessment of RR manually is a limitation of this study, considering that it is usually the least documented vital sign strongly related to measurement errors;^(25)^ in this sense, the number of RRT activations might be impaired. The unicentric characteristics and the small sample size are other limitations of this protocol. Considering that we had no knowledge of the intraclass correlation coefficient to make the appropriate formal calculation for the cluster design, the sample size calculation was only a preliminary estimate, characterizing this study as exploratory; on the other hand, statistical analyses should adjust for the baseline values of RRTs in the participating wards, which should reduce the random error of the estimates. Additionally, the selection of the primary outcome was made taking into account the exploratory design of this trial. We hope that from the results obtained, it will be possible to carry out a multicenter study with greater coverage so that we can confirm our findings. Another potential limitation is performance bias, since it is an open-label study because of the nature of the intervention. However, all other procedures involving the management of patients in the wards will be maintained at the discretion of the medical and nursing teams involved in the assistance. In addition, we chose objective outcomes, and the team performing data collection and statistical analysis will be blinded to the participant’s group, minimizing the effects of this potential bias on the results.

The recruitment of the study was completed in September 2021. Currently, the Hillrom study is in the data collection phase, identifying RRTs that have been properly triggered. The study is expected to end in August 2022.
